# Melanocortin Peptide Therapy for the Treatment of Arthritic Pathologies

**DOI:** 10.1100/tsw.2009.163

**Published:** 2009-12-16

**Authors:** S. J. Getting, M. Kaneva, Y. Bhadresa, D. Renshaw, Giovanna Leoni, H. B. Patel, M. J. P. Kerrigan, I. C. Locke

**Affiliations:** ^1^ Inflammation and Infection Group, School of Life Sciences, University of Westminster, London, UK; ^2^ Centre for Biochemical Pharmacology, William Harvey Research Institute, London, UK

**Keywords:** melanocortin, neutrophil, migration, anti-inflammatory, macrophage, rheumatoid arthritis, gout, osteoarthritis, cytokine, chemokine

## Abstract

Arthritic pathologies are a major cause of morbidity within the western world, with rheumatoid arthritis affecting approximately 1% of adults. This review highlights the therapeutic potential of naturally occurring hormones and their peptides, in both arthritic models of disease and patients. The arthritides represent a group of closely related pathologies in which cytokines, joint destruction, and leukocytes play a causal role. Here we discuss the role of naturally occurring pro-opiomelanocortin (POMC)-derived melanocortin peptides (e.g., alpha melanocyte stimulating hormone [a-MSH]) and synthetic derivatives in these diseases. Melanocortins exhibit their biological efficacy by modulating proinflammatory cytokines and subsequent leukocyte extravasation. Their biological effects are mediated via seven transmembrane G-protein-coupled receptors, of which five have been cloned, identified, and termed MC_1_ to MC_5_. Adrenocorticotrophic hormone represents the parent molecule of the melanocortins; the first 13 amino acids of which (termed a-MSH) have been shown to be the most pharmacologically active region of the parent hormone. The melanocortin peptides have been shown to display potent anti-inflammatory effects in both animal models of disease and patients. The potential anti-inflammatory role for endogenous peptides in arthritic pathologies is in its infancy. The ability to inhibit leukocyte migration, release of cytokines, and induction of anti-inflammatory proteins appears to play an important role in affording protection in arthritic injury, and thus may lead to potential therapeutic targets.

## THE ARTHRITIDES

The term “arthritides” refers to a wide range of musculoskeletal disorders that affect a large number of individuals worldwide. In the U.K. alone, musculoskeletal disorders are the most prevalent group of limiting long-term illness, representing 34% of all such illnesses reported, with heart and circulatory disease the next most prevalent at 19%[1]. It has been estimated that the annual cost of treatment for musculoskeletal disorders is between 1 and 2.5% of the gross national product of countries like the U.K.,

U.S., Canada, France, and Australia[2]. Three of the most common arthritides are osteoarthritis (OA), rheumatoid arthritis (RA), and metabolic arthritis, also known as gouty arthritis.

## Osteoarthritis

OA is the most common of the arthritides, and is predominantly a disease of the elderly and middle aged. It is a degenerative disease characterised by the loss of articular cartilage, resulting in a loss of joint mobility and pain. Although it can appear in any synovial joint, OA usually manifests in the larger joints first, with the hip, knee, spine, and, later, hand being commonly affected[3]. Whilst the exact pathogenesis of OA is not fully understood, the disease appears to be largely a result of long-term or abnormal cartilage “wear and tear”, coupled with a reduction in extracellular matrix (ECM) maintenance and turnover by the chondrocytes as a result either of chondrocyte death by apoptosis or chondrocyte senescence[4,5]. Changes in chondrocyte phenotype resulting in increased levels of catabolic enzymes, such as the matrix metalloproteinases (MMPs), have also been suggested to play a role in the cartilage destruction observed in OA. A variety of chemical stimuli appears to promote chondrocyte apoptosis, including proinflammatory cytokines, nitric oxide (NO), and Fas ligand (FasL). Other possible cell death inducers include lack of nutrients (e.g., amino acids, glucose), hypoxia, mitochondrial damage, and ECM acidosis caused by lactate accumulation[6].

## Rheumatoid Arthritis

RA is the most common of the arthritides to which a causal inflammatory component can be attributed. RA can affect males and females of any age group, but there is a marked female bias (approximately 2.5 times) and the disease is most commonly seen in middle-aged individuals. In similarity to OA, RA can also appear in any synovial joint, but conversely to OA, RA usually manifests first as a symmetrical inflammation of the smaller joints, with the hands and feet being most frequently involved. Like OA, the exact pathology of RA is still largely unknown, but it is generally accepted to be a disease of autoimmune origin resulting in a persistent immunological reaction that is largely directed against joint tissues, but may also manifest as a more systemic syndrome involving a variety of organs. Characteristic findings in the RA joint include synovial inflammation with an accumulation of inflammatory cells, increased synovial levels of proinflammatory cytokines, and the formation of a characteristic area of “granulation tissue” known as a pannus, which is formed from both synovial cells and the cellular infiltrate, and appears to contribute to the destruction of the underlying cartilage[7]. Whilst RA is generally classified as an autoimmune disease, the mechanisms involved and the significance of the autoantibodies routinely detected in RA patients is uncertain. The main autoantibodies, detected in approximately 70–80% of RA patients, are rheumatoid factor (RF), an antibody directed against the fragment crystallisable (Fc) region of IgG antibodies, and anticyclic citrulinated peptide (anti-CCP) antibodies, which are directed against proteins/peptides containing citrulline, a modified form of the amino acid arginine. Although the contribution of these antibodies to the disease process remains uncertain, it is proposed that they may be involved via the formation of immune complexes, with their target antigens resulting in complement activation. The activation of complement, along with the release of other proinflammatory mediators from the synovium, may act in turn as a driving force for leukocyte recruitment and infiltration of the joint space[7,8,9].

## Metabolic Arthritis/Gouty Arthritis

Like RA, gouty arthritis is also an inflammatory arthritis that predominantly affects middle-aged/older individuals, but unlike RA, gout has a marked male bias (approximately 5 times). Gouty arthritis can occur in any synovial joint, but most commonly manifests as a monoarthritic attack in the smaller joints and, in particular, the first metatarsophalangeal joint of the big toe, resulting in symptoms including erythema, oedema, and severe pain. Gouty arthritis occurs largely as a result of hyperuricaemia (increased levels of plasma urate, a product of purine metabolism), although other factors (such as joint trauma and local temperature) may also play a role. When plasma solubility of urate is exceeded, crystals of monosodium urate (MSU) become deposited in a variety of soft tissues and the joints, where it results in gouty arthritis. Whilst the mechanisms of urate-induced inflammation are not fully elucidated, gouty arthritis seems to be initiated primarily by an innate immune response, chiefly involving cells of the monocyte/macrophage lineage and neutrophils. One of the cardinal features seen in a joint affected by gouty arthritis is a major infiltration of neutrophils resulting from increased expression of endothelial cell adhesion molecules mediated by proinflammatory mediators, such as interleukin (IL)-1β, tumour necrosis factor (TNF)-α, and NO. Recent work has shown that MSU crystals are recognised by the TLR2 and TLR4 toll-like receptors present on phagocytes and other cells within the joint environment, resulting in the generation of these proinflammatory mediators and, hence, providing a possible mechanism for the disease process[10,11].

## Current Therapies

Currently, disease-modifying antirheumatic drugs (DMARDS), nonsteroidal anti-inflammatory drugs (NSAIDs), TNF-α inhibitors, and glucocorticoids are routinely used therapeutically in the treatment of the arthritides in general and RA in particular (Table 1). However, a large number of patients can be nonresponsive[12].

**TABLE 1**
**Therapeutic Treatments for Arthritic Patholgies**

| Medication Type | Examples |
|---|---|
| DMARDs | Azathioprine, chloroquine, cyclosporine, hydroxychloroquine, leflunomide, methotrexate, auranofin, sodium aurothiomalate, penicillamine |
| NSAIDs | Aspirin, diclofenac, ibuprofen, indomethacin, meloxicam, piroxicam, |
| COX-2 specific NSAIDS (“coxibs”) | Celecoxib, etoricoxib, lumiracoxib, |
| Glucocorticoids | Hydrocortisone, prednisolone, triamcinolone |
| Biologic response modifiers | |
| TNF-α inhibitors | Adalimumab, etanercept, infliximab |
| IL-1 receptor antagonists | Anakinra |
| T-cell costimulatory blocking agents | Abatacept |
| B-cell depleting agents | Rituximab |

Alongside these established treatments, however, several novel lines of approach are currently being investigated for the treatment and management of arthritic pathologies. Some of these therapies are as follows:

1. RA-targeting TLR4 and/or tenascin-4 in murine models. Mice not expressing tenascin-4 showed a rapid resolution of inflammation and protection from erosive arthritis[13].
2. RA-small interference RNA–targeting CD81 in a rat model of arthritis led to a reduction in hypertrophy of synovium, bone erosion, and degeneration of articular cartilage[14].
3. Gout-targeting glucose transporter 9 (GLUT9) may reduce gout since it is expressed in articular cartilage and known to be a uric acid transporter[15].

4. Gout-targeting IL-1 Trap in a murine ankle joint model of urate crystal deposition led to reduction in inflammation[16].
5. OA inhibition of IL-1β–converting enzyme reduced joint damage[17].
6. OA antagonism of prostanoid EP4 led to a reduction in the signs and symptoms of OA[18].

Another line of interest in the treatment of these diseases is the identification of endogenous pathways that control the host inflammatory response. The identification of the mediators involved and their target receptors may lead to novel therapeutic targets, with the development of compounds that may have fewer side effects. In this view, identification of novel antiarthritic drugs with minimal side effects needs to remain in the forefront of arthritis research. The potential anti-inflammatory role for endogenous peptides was first demonstrated in the late 1940s, whereby clinical studies performed by Nobel Prize–winner Philip Hench showed that the use of the pituitary hormone adrenocorticotrophin (ACTH) resulted in a beneficial effect in patients with severe RA. Each patient received 100 mg of ACTH, with considerable improvement in joint pain, swelling, and rheumatic symptoms[19,20] similar to that seen with corticosterone therapy. However, treatment with ACTH was not without consequence, with some patients showing side effects associated with hypothalamic-pituitary-adrenal (HPA) axis stimulation. Despite this beneficial outcome, the exact mechanism through which ACTH mediated this antiarthritic effect was never fully investigated. This review will focus on the beneficial effects of melanocortin peptides derived from the pro-opiomelanocortin (POMC) gene.

## PRO-OPIOMELANOCORTIN PROTEIN

Melanocortins are ancient peptides derived from the POMC gene that are little changed throughout evolution and can be traced back over 700 million years[21] to the appearance of the first vertebrates, showing similarities to molecules present in lampreys and gnathostomes[22]. Over 50 years ago, following the initial identification of their involvement in skin pigmentation, these peptides were also shown to modulate many disease pathologies[23].

The POMC gene is 241 amino acids in humans[24], and 209 in mouse[25] and rat[26]. The POMC contains three main domains: the N-terminus region, which contains γ-MSH (melanocyte stimulating hormone); the central highly conserved $ACTH_{1-39}$ sequence, with α-MSH at its N-terminus; and the C-terminal β-lipotropin, which can be cleaved to generate β-endorphin[27] (Fig. 1). These peptides are formed following post-translational processing by prohormone converting (PC) enzyme and carboxypeptidases cleaving between two pairs of basic amino acid residues (-Lys-Lys, -Arg-Lys-, -Arg-Arg-, -Lys-Arg-). Seven members of the PC family exist, including PC1/3, PC2, furin/PACE, PACE4, PC4, PC5/6, and PC7/SPC7/LPC/PC8[28], with PC1 cleaving the POMC protein into $ACTH_{1-39}$ and β-lipotropin, together with low concentrations of β-endorphin, whilst PC2 cleaves POMC protein into β-endorphin and β-MSH. The existence of β-MSH has been questioned and it has been suggested that it may be an artefact of the β-lipotropin molecule formed following certain extraction procedures[29,30], due to the fact that the pars intermedia present in other species does not exist.

## MELANOCORTIN RECEPTORS: BINDING AND DISTRIBUTION

Melanocortins exert their biological effects by binding to the shortest family of seven transmembrane G-protein-coupled receptors (GPCRs), since they have a small second extracellular loop and short amino and carboxy terminal end[31]. The melanocortin receptor (MC) family consists of five members that have been cloned and termed $MC_1$ to $MC_5$. Each receptor is positively coupled to adenylylate cyclase, with activation leading to cyclic adenosine monophosphate (cAMP) accumulation following ligation by the common amino acid sequence His-Phe-Arg-Trp (HFRW).

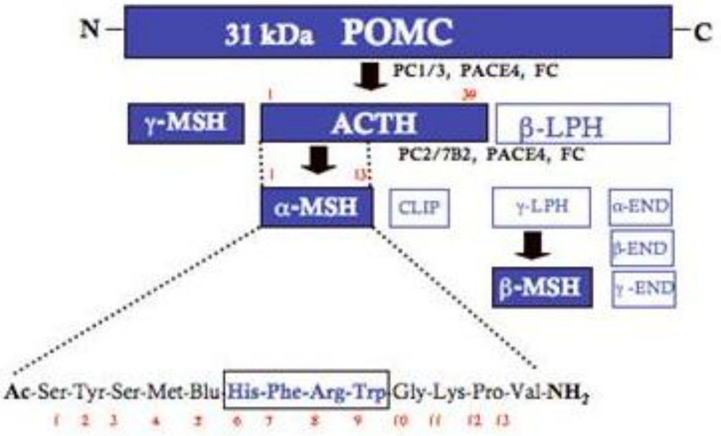

**FIGURE 1.** Schematic representation of POMC gene and its products. Post-transcriptional processes of POMC induce the production of ACTH, α-, β-, and γ-MSH. The figure shows also the amino acid sequence of α-MSH.

MCs have been shown to be expressed in a multitude of tissues[31] with varying levels of sequence homology between the different receptors, e.g., 38% similarity between $MC_2$ and $MC_4$ and 60% between $MC_4$ and $MC_5$. In addition to elevations in cAMP, melanocortins such as α-MSH can down-regulate nuclear factor kappa B (NF-κB) activation (by preventing phosphorylation and degradation of inhibitors of kappa B [IκB]) and thus cytokine synthesis[32,33]. Increases in intracellular calcium and secondary activation of inositol triphosphate have also been proposed[34]. Other GPCR-associated recognition sites have also been identified on the melanocortins, including those for protein kinase (PK) C and/or PKA[35]. A number of natural and synthetic melanocortin peptides have been isolated and shown to bind to the five MCs with different efficacies. These are briefly discussed below, but for a more detailed analysis please refer to this excellent review[31].

- **$MC_1$** — $MC_1$ is a 317-amino-acid protein cloned in 1992[35,36]. This receptor is involved in numerous functions, including pigmentation, and antipyretic and anti-inflammatory actions. It is expressed by a number of cell types, including melanocytes, monocytes[37], neutrophils[38], endothelial cells[39], fibroblasts[40], mast cells[41], and lymphocytes[42]. In addition to this peripheral expression, $MC_1$ has also been detected in the brain[43]. The receptor is activated by a number of endogenous peptides, with α-MSH being the most active followed by $ACTH_{1-39}$, with β-MSH and γ-MSH causing weak activation[23]. Truncated peptides $ACTH_{4-10}$ and $ACTH_{1-10}$ do not activate $MC_1$, suggesting that both the amino and carboxyl-terminal ends of α-MSH ($ACTH_{1-13}$) are important for full biological activation[44].
- **$MC_2$** — $MC_2$ is a 297-amino-acid receptor activated only by $ACTH_{1-39}$, with no biological efficacy attributed to other melanocortin peptides. The receptor is involved with regulation in the release of steroids by the adrenal cortex essential for steroidogenic enzymes[45], and is detected in the zona glomerulosa and fasiculata areas of mineralcorticoid and glucocorticoid production[36,46].
- **$MC_3$** — $MC_3$ is expressed in both the periphery and the central nervous system (CNS)[47]. $ACTH_{1-39}$, α, β, and γ-MSH all activate the receptor with equal potency, and indeed the truncated peptides $ACTH_{4-10}$ and $ACTH_{1-10}$ are also fully efficacious in contrast to their effects on $MC_1$[47]. $MC_3$ has been proposed to be involved in modulating energy metabolism[48] and a role in modulating inflammatory disease has also been reported[49,50,51]. The latter is suggested by the observation that activation of $MC_3$ leads to an initial reduction in proinflammatory cytokines and chemokines, followed by the induction of anti-inflammatory mediators at later time points[52].

Studies have also highlighted a protective effect of melanocortins in ischaemia-reperfusion (IR) injury[53,54]. This may suggest that $MC_3$ could be a “fine tuner” of specific mechanisms involved during energy metabolism, inflammatory disease, and cardiovascular function.

- **$MC_4$** — $MC_4$ is a 332-amino-acid protein with a 93% sequence homology between rat and human[55], and is expressed solely within the brain, including the hypothalamus, spinal cord, and cortex[56]. Receptor activation is similar to $MC_1$, with $ACTH_{4-10}$ and $\gamma$-MSH inactive due to the absence of $PRO^{12}$, a prerequisite for $MC_4$ binding[57]. Potential roles in obesity, erectile dysfunction[58], and pain[59] have been proposed for this receptor.
- **$MC_5$** — $MC_5$ is 325 amino acids and is expressed solely in the periphery, including the liver, lung, and testis. In addition, its expression has been detected in cells of the immune system, including B[60] and T lymphocytes[61], suggesting a role in immune regulation. A role in exocrine sebaceous gland secretion has also been proposed[62]. Peptides activate this receptor in a similar fashion to that of the $MC_1$; however, $ACTH_{1-10}$ and $ACTH_{4-13}$ have full activity, albeit with a greatly diminished potency compared to $\alpha$-MSH[63].

## MELANOCORTIN PEPTIDES

Synthetic and nonpeptide mimetic development has enhanced our understanding of the biological functions of MCs in different tissues. High sequence homology between receptors, however, has hindered the development of selective compounds and has caused problems in unravelling their physiological roles. Endogenous and synthetic peptides have been utilised to evaluate the pathophysiological role of the various MCs in different tissues. The minimum fragment identified for activation of the MCs is the tetrapeptide $ACTH_{6-9}$ (HFRW)[34]. A number of peptides and nonpeptide compounds have been developed over the last 20 years with varying degrees of selectivity to help elucidate the roles played by each receptor in disease pathologies. This has proved problematic, however, due to high sequence homologies between the five MCs[64]. Highlighted below are some of the main peptides used within the field of melanocortin pharmacology (Fig. 2).

**ACTH**
Ser-Tyr-Ser-Met-Glu-**His-Phe-Arg-Trp**-Gly-**Lys-Pro-Val**- Gly-Lys-Lys-Arg-Arg-Pro-Val-Lys-Val-Tyr-Pro-Asn-Gly-Ala-Glu-Asp-Glu-Ser-Ala-Glu-Ala-Phe-Pro-Leu-Glu-Phe

**$\alpha$-MSH:**
Ac-Ser-Tyr-Ser-Met-Glu-**His-Phe-Arg-Trp**-Gly-**Lys-Pro-Val**

**$\beta$-MSH:**
Asp-Glu-Gly-Pro-Tyr-Arg-Met-Glu-**His-Phe-Arg-Trp**-Gly-Ser-Pro-Pro-Lys-Asp

**$\gamma_1$-MSH:**
Tyr-Val-Met-Gly-**His-Phe-Arg-Trp**-Asp-Arg-Phe-Gly

**$\gamma_2$-MSH:**
Tyr-Val-Met-Gly-**His-Phe-Arg-Trp**-Asp-Arg-Phe-Gly

**$\gamma_3$-MSH:**
**Tyr-Val-Met-Gly-His-Phe-Arg-Trp**-Asp-Arg-Phe-Gly-Phe-Arg-Asn-Ser-Ser-Ser-**Ala-Gly-Ser-Ala-Ala-Gln-Arg-Arg**

**FIGURE 2**. Sequences of melanocortin peptides. All peptides contain the common amino acid motif sequence (in bold), and ACTH with $\alpha$-MSH also has in common the tripeptide KPV (highlighted in red).

## Agonists

[$Nle^4$,D-$Phe^7$]-α-MSH (NDP-α-MSH) was one of the first synthesised analogues of α-MSH to display efficacy in inflammatory models[65]; however, due to its length, the economic reality of bringing this peptide to market led to the development of smaller compounds. Given the short half-life and size of the peptide, smaller fragments were developed, including the potent $MC_3/_4$ cyclic heptapeptide agonist melanotan II (MTII), (Ac-$Nle^4$-c[$Asp^5$,D-$Phe^7$,$Lys^{10}$]α-MSH (4-10)-$NH_2$)[66], shown to be effective in models of murine obesity[67] and gouty arthritis[49,50,51]. Further identification of the natural agonists of the $MC_3$ led to the discovery that Trp(8) substitution into the natural sequence of γ-MSH led to a selective compound termed $dTRP^8$-γ-MSH[68]. This compound has a 300- and 250-fold increase in selectivity for the $MC_3$ over the $MC_4$ and $MC_5$, respectively[68]. Grieco and colleagues have also been instrumental in altering the chemical structure of the $MC_3/_4$ antagonist SHU9119 (see below) by substituting His(6) with Pro(6) or Hyp(6), leading to full agonists at the $MC_5$; these compounds have been termed PG901 and PG911[68]. These findings highlighted that those subtle changes of amino acids at discrete positions within the α-MSH sequence lead to the development of increasingly more selective compounds. Development of selective $MC_1$ agonists MS05 and MS09 occurred following screening of peptide phage display library[69], with MS09 being more potent than MS05, but less selective[34]. Both these compounds have been shown to down-regulate the expression and secretion of endothelial cell selectin (E-selectin), vascular cell adhesion molecule (VCAM), and intracellular endothelial cell adhesion molecules (ICAM) in human dermal vascular endothelial cells treated with TNF-α, and also down-regulate TNF-α–induced NF-κB activation in these cells[70]. In a murine model of TNF-α–induced secretion, the octapeptide 154N-5, which shows a high degree of selectivity for human and mouse $MC_1$, inhibited TNF-α secretion[71].

## Antagonists

Development of SHU9119 was one of the major breakthroughs in melanocortin pharmacology. Substitution of a bulky aromatic amino acid D-Nal(2) into position 7 of $ACTH_{1-39}$ leads to an antagonist at $MC_3$ and $MC_4$ with the structure Ac-$Nle^4$-c[$Asp^5$,D-$Nal(2)^7$,$Lys^{10}$]α-MSH (4-10)-$NH_2$[72]. SHU9119 has allowed many groups to dissect the role of the $MC_3$ in diseases ranging from arthritis to cardiovascular pathologies, and also in models of obesity. This compound has been shown to be an antagonist at $MC_3/_4$ in a mouse model of obesity[67] and in models of acute inflammation[39,40,41,42,43,44,45,46,47,48,49,50,51]. Other substitutions into positions 6 to 15 of $ACTH_{1-39}$ have yielded the $MC_4$ antagonist HS014[64], which blocked cAMP induced by α-MSH in $MC_4$-transfected COS-1 cells. Whilst mentioned previously as an agonist, Grieco and colleagues also found that alteration of SHU9119 by substituting His(6) with Pro(6) or Hyp(6) to generate full $MC_5$ agonists (PG901 and PG911) also results in these peptides being full antagonists at the human $MC_3$ and $MC_4$[68]. The selective cyclic $MC_4$ antagonist HS024[73] has an enhanced 10-fold potency compared to HS014[64] and has been demonstrated to stimulate food intake in rats[73]. In addition to these synthetic antagonists, two naturally occurring ones exist: the Agouti signalling protein (ASP), a 131-amino-acid protein found in rodents and only expressed in the skin[31,74], and human ASP, which is closely homologous to the rodent agouti. Human ASP, however, is more widely distributed, with expressions in the foreskin, liver, kidney, adipose tissue, heart, testis, and ovary[31,75,76]. ASP has been shown to inhibit the binding of α-MSH to $MC_1$ and $MC_4$ competitively[31,74] and thus may be important in inflammation[31]. A further protein, the Agouti-related protein (AGRP), is specifically expressed in the CNS, in particular the neuronal cell bodies of the posterior hypothalamus[31], and has been demonstrated to be an antagonist of $MC_3$[77,78]. In a murine model of urate crystal peritonitis, AGRP blocked the anti-inflammatory effects of the selective $MC_3$ agonist $dTRP^8$-γ-MSH[79].

In addition to these amino acid substitutions of naturally occurring melanocortin peptides, the pharmaceutical industry has started to develop nonpeptidic ligands that should have improved potency, selectivity, and bioavailability[80]. THIQ[81] and MB243 developed by the Merck group are $MC_4$ agonists, with MB243 shown to have a 100-fold selectivity for the $MC_4$ compared to $MC_{1,\ 3,\ 5}$[82], and demonstrating potential in erectile dysfunction therapy[83,84]. Neurocrine Biosciences of California have also developed analogues of piperazinebenzylamines with nanomolar activity at human $MC_4$, termed 14C, which when injected i.c.v. resulted in a potentiation of food intake in satiated mice[85]. Similarly, a 2-ethoxycarbonylcyclohexyl-piperazines derivative, 12i, displayed oral bioavailability in rats and significantly increased food intake in mice[86]. Bristol-Myers Squibb has also developed an $MC_1$ small molecule agonist termed BMS470539, which they demonstrated to be efficacious in a model of acute inflammation[87].

## MELANOCORTINS AND CELL SIGNALLING

MC activation positively stimulates adenylylate cyclase with subsequent elevation in intracellular cAMP[34]. In addition, agonistic effects of melanocortin peptides on a number of pathways have been postulated, including p38[88], extracellular signal regulated kinases (ERK) (p44/42)[89], PKC[90], and nuclear transcription factors AP-1[91] and NF-κB[92,93,94].

α-MSH has been shown to regulate NF-κB in the immune system by preventing degradation of IκBα elicited by agents such as lipopolysaccharide (LPS) and TNF-α in human monocytic and macrophage cell lines. This appears to occur via a cAMP-dependent PKA pathway, resulting in the subsequent inhibition of NF-κB translocation and gene transcription[33], including a reduction in the array of cytokines and chemokines (including TNF-α and IL-8), adhesion molecules, and inducible enzymes involved in inflammation (NOS-2 and COX-2)[92]. This protective effect of α-MSH is not solely restricted to monocytic cells with similar observations made in human microvascular endothelial cells (HMEC-1), where α-MSH prevents LPS-induced VCAM-1 and E-selectin up-regulation following NF-κB activation[93]. A possibility for gene therapy in the treatment of chronic inflammatory lung diseases has been suggested, where plasmid vector encoding α-MSH can lead to autocrine α-MSH inhibiting LPS-induced NF-κB activation following preservation of expression of IκBα in A549 epithelial cells[94]. It was further shown that transfection of the epithelial cell lines suppressed expression of an NF-κB–dependent reporter gene, supporting an inhibitory role of α-MSH on this transcription factor.

In addition to the well-reported role of α-MSH in NF-κB regulation, studies have also highlighted a role for melanocortins in the regulation of melanogenic and proliferative responses via cAMP and mitogen-activated protein kinase (MAPK) pathways. In B-16 melanoma cells, NDP-α-MSH activation leads to an increase in cAMP and subsequent phosphorylation of $p44^{mapk}$ via a Raf-1–independent MEK cascade. Phosphorylated $p44^{mapk}$ then translocates to the nucleus to stimulate AP1 (nuclear transcription factor), possibly through the phosphorylation of JunD or up-regulation of Fra-2, resulting in the augmentation of tyrosinase mRNA, a rate-limiting enzyme required for melanogenesis[91]. In partial agreement with this study, α-MSH caused a time-dependent phosphorylation of both $p44/42^{mapk}$ and p38 in B-16 melanoma cells, but only the inhibition of p38 activity resulted in the abrogation of the PKC-dependent α-MSH–induced melanogenic and antiproliferative effects[88]. The ability of melanocortin peptides to induce anti-inflammatory cytokines in a murine macrophage cell line (RAW264.7) has also been investigated. Stimulation with $ACTH_{1\text{-}39}$ or the $MC_3/_4$ agonist MTII did not alter ERK1/2 and JNK phosphorylation, whilst p38 phosphorylation and intracellular cAMP accumulation occurred within minutes. Cell incubation with $ACTH_{1\text{-}39}$ and MTII provoked a time-dependent accumulation of IL-10 that was abrogated by the PKA inhibitor H-89, and only partially blocked by the p38 MAPK inhibitor SB203580[95] and the anti-inflammatory protein heme oxygenase 1 (HO-1)[96]. Future investigation into the role of MAPK in MC signal transduction is required in order to determine the part played by these enzymes in specific receptor-mediated responses.

In human $MC_3$-transfected HELA cells, activation of cAMP-dependent PKA in the presence of α-MSH was shown to exert an inhibitory effect on the $IP_3$-mediated increase in intracellular $Ca^{2+}$ concentration[95]. In addition, other postreceptor signal transduction events reported include signal transduction via the Jak/STAT pathway (an intracellular phosphorylation mechanism used by cytokines and growth factors) in α-MSH–stimulated $MC_5$ on B-lymphocytes[60], activation of tyrosine kinase and PKC in α-MSH–treated rat adrenal zona glomerulosa in steroidogenesis[93], and the enhancement of eicosanoids by α-MSH in retinal pigment epithelium[97].

## ROLE OF MELANOCORTINS IN DISEASE PATHOLOGIES

In this review, we will discuss the role of melanocortins in inflammatory disease; in particular, arthritic pathologies. However, we would like to refer the readers to a number of other reviews and papers that highlight the multiple biological effects of these peptides that are not within the scope of this review. These areas cover (1) skin physiology and melanocyte function[98,99]; (2) pain and nerve regeneration[100,101,102]; (3) behaviour, learning, and memory[103,104,105]; (4) obesity and energy metabolism[106,107,108]; (5) allergic inflammation[109,110,111,112,113], (6) mesenteric ischaemia and reperfusion[114,115]; (7) cardiovascular disease[54].

### Role of Melanocortin in Inflammatory Disease

Melanocortins have been shown to exhibit a multitude of anti-inflammatory effects in *in vitro* and *in vivo* disease models restoring homeostasis to tissue; thus, identification of highly selective peptides and small molecule inhibitors will aid in the therapeutic development of melanocortin-based treatments. Seminal studies by Lipton’s group evaluated the potential of α-MSH in fever, demonstrating a potent antipyretic effect in rabbits injected with endogenous pyrogen[116]. This protective effect was attributed to the inhibition of proinflammatory cytokines, including IL-1[116], IL-6[117], and TNF-α[113,117]. Other studies further support the role played by α-MSH in modulating fever, demonstrating that the injection of a highly specific antiserum against α-MSH results in prolonged fever[118].

A role for melanocortin treatment in bronchial asthma, characterised by an infiltration of eosinophils into the airways[119,120], has also been proposed with these studies highlighting the potential use of melanocortins in controlling airway inflammation. Using an allergic model of lung inflammation, Raap and colleagues demonstrated that α-MSH inhibited eosinophil migration into the airways with subsequent reduction in IgE, IL-4, and IL-13, but no effect was observed on IL-5 even though eosinophil numbers were reduced. These observations may be explained by α-MSH inhibition of VCAM-1, which is under the control of IL-4 and IL-13[120]. These protective effects of α-MSH appear to involve its ability to induce the anti-inflammatory cytokine IL-10, since the anti-inflammatory effects of α-MSH were not observed in IL-10 null mice[120]. In addition to these antimigratory and anticytokine effects, α-MSH demonstrated an ability to modulate the underlying pathology of bronchial hyper-responsiveness (BHR)[120] and may thus have similar effects to steroids, but potentially without the side effects.

The ability to inhibit eosinophil and lymphocyte migration in a model of allergic inflammation by the selective $MC_3$ agonist $dTRP^8$-γ-MSH and by the pan agonist α-MSH, but not the $MC_1$-selective agonist MS05[119], suggests a role for the $MC_3$ in these pathologies. This is further supported by studies using genetically modified mice (namely, the recessive yellow e/e mouse, which has a nonfunctional $MC_1$[51], and the $MC_3$ null mouse[121]), where α-MSH retains its anti-inflammatory role in recessive yellow e/e mice, but not the $MC_3$ null mouse[119]. This study also demonstrated that melanocortins possess the ability to modulate nonallergic LPS-induced lung inflammation by inhibiting neutrophil accumulation and inhibition of TNF-α release in two different models of lung inflammation. It was further found that this was due in part to inhibition of TNF-α in the nonallergic model of inflammation, but not IL-5 inhibition in the allergic model. In experimental models of colitis that display characteristic signs of human inflammatory bowel disease, including markers such as weight loss and faecal blood, the pan agonist α-MSH has been shown to be effective in controlling the inflammation associated with this disease. In murine models, α-MSH induces the anti-inflammatory cytokine IL-10, thus promoting the resolution of inflammation, a protective effect lost in IL-10 null mice[120]. In the dextran sulfate colitis model, α-MSH exhibits a protective effect against these markers described above and, in addition, inhibits TNF-α and NO production[122]. Further studies using the trinitrobenzene sulfonic acid–induced colitis model also reveal similar protective effects with α-MSH, which in this case appear to be dependent on NO and prostaglandin generation, since α-MSH effects were blocked by sodium nitroprusside and indomethacin[123]. Therapeutic treatment of mice with recombinant *Lactobacillus casei*, which secretes α-MSH, displays significant anti-inflammatory effects in dextran sulfate–induced colitis[124]. These effects occurred via oral administration and thus open a new avenue for the delivery of these peptides to sites of inflammation. Recent studies have focused on the α-MSH C-terminal region KPV, which displays potent anti-inflammatory properties in gouty arthritis[125] and two murine models of colitis where it demonstrated a protective effect in a partially $MC_1$-independent manner[126]. These observations in preclinical models of intestinal disease have been translated to humans, where patients with celiac disease have immunoreactivy for α-MSH, $MC_1$, and $MC_5$[127].

## Role of Melanocortins in the Arthritides

### *Rheumatoid Arthritis*

RA is a complex pathology affecting many systems outside of the joints, with 40% of RA deaths due to cardiovascular disease. These are likely to be a result of several risk factors, including high blood pressure, diabetes, high cholesterol, and obesity. Therapeutic intervention in RA can also be added to these complications. NSAIDs used, e.g., ibuprofen, may contribute to raised blood pressure. Glucocorticoids, e.g., prednisone, can also increase cardiovascular problems by accelerating the rate of pathologies such as arterial thickening and narrowing. Of the commonly used DMARDS, methotrexate may also promote heart disease by increasing levels of homocysteine. Given that these conventional therapies employed in RA can lead to cardiovascular problems and that NSAIDs are contraindicated in the elderly due to decreased renal clearance, the development of novel endogenous therapeutics is essential.

To date, the role of melanocortin peptide(s)/receptor(s) in RA is uncertain. Catania and colleagues identified elevated levels of α-MSH in the synovial fluid, but not the plasma, of patients with RA and juvenile chronic arthritis, but not in OA patients[128]. This increase in α-MSH within synovial fluid could indicate local production within the joint, and suggests the possibility of an endogenous anti-inflammatory loop to maintain a homeostatic balance within the joint and control the host inflammatory response. Joint concentrations of α-MSH in these patients were directly proportional to the degree of inflammation, whilst systemic (plasma) levels remained at physiological concentrations[128].

Given the nature of the cellular milieu present in RA joints, it seems likely that monocytes and/or macrophages, which are known to synthesise melanocortin peptides[129], could be an important source. Utilising the preclinical adjuvant-induced arthritis model, α-MSH was shown to modulate weight loss, arthritic score, joint damage, and swelling, features all observed in patients with this disease. Of further interest were the observations that α-MSH had greater beneficial effects in this model than those observed with the glucocorticoid prednisolone, including preventing glucocortiocid-induced weight loss[130]. The inflamed joint is characterised not only by infiltrating leukocytes, but also by activated resident bone/cartilage cells, such as osteoclasts and chondrocytes. Some evidence exists to suggest MC

expression on these cells[131]. *In situ* hybridisation has shown all the MCs to be expressed on chondrocytes in the mouse femoral bone and mRNA signals for all MCs, except $MC_1$, were detected on primary rat osteoclasts, which, interestingly, also expressed the POMC gene[132]. These data suggest the possibility that POMC peptides generated by these cells could act in an autocrine/paracrine manner through their corresponding MCs. α-MSH has been shown in a treatment of a chondrocyte cell line to be able to inhibit MMP-13, an enzyme known to be involved in ECM degradation in healthy and diseased cartilage[132,133]. These clinical findings suggested that α-MSH may be beneficial in treating arthritic conditions.

## Osteoarthritis

OA is a chronic disorder characterised by damage to cartilage and tissue that leads to a loss of joint function, pain, bone overgrowth, and stiffness, damage that generally increases with age. Current treatment regimes for OA largely focus on exercise, surgery for joint replacement, and the treatment of symptoms, e.g., relieving pain with analgesics, rather than treating the underlying causes of the disease. Only a handful of studies so far have evaluated the therapeutic potential of ACTH and melanocortin peptides in OA. This may seem somewhat surprising at first glance given the number of studies suggesting therapeutic potential of these peptides in both RA and gouty arthritis. However, this could stem from the fact that whilst inflammation is considered causal to both RA and gouty arthritis, it is not generally accepted to be a major contributor to the development of OA.

Catania and colleagues evaluated the presence of systemic and synovial α-MSH in patients with OA and RA to evaluate whether changes in levels correlated with disease progression. α-MSH was detected in the synovial fluid of both OA and RA patients, with levels that were much lower in OA than RA. Of interest, however, was the observation that synovial levels of α-MSH were higher than plasma, suggesting a local production of this peptide[128]. This could indicate that activation of resident cells within the joints causes the release of α-MSH, which in turn switches off disease progression in an auto or paracrine fashion. Again, given that inflammation is only considered to be a component of OA rather than a causal factor, this could explain why much lower levels of α-MSH are found in OA rather than RA patients.

One potential mechanism for the articular cartilage degradation observed in OA may be the TNF-α–induced expression of MMPs. As mentioned earlier, α-MSH can down-regulate TNF-α–induced expression of MMP-13 mRNA and protein in the human chondrosarcoma cell line HTB-94 (SW1353)[133]. Studies with the pharmacological inhibitor SB203580, a p38 MAPK inhibitor, showed that α-MSH inhibited MMP-13 by modulating p38 MAPK phosphorylation and subsequent activation of NF-κB[134]. This agrees in part with previous studies in the murine cell line RAW264.7, where $ACTH_{1-39}$ and the $MC_3/_4$ agonist MTII accumulation of IL-10 were also partially blocked by SB203580[96].

Although α-MSH has been shown to decrease TNF-α–induced MMP expression, the parent hormone ACTH can cause terminal differentiation of chondrocytes and subsequent cartilage degeneration. Following cartilage degradation, bone can overgrow at the edges of joints, causing the cartilage to become rough and pitted, and the joint can no longer move smoothly. Using rodent chondrocytes and chondrocyte cell lines that have been shown by western blotting analysis to express the $MC_3$, matrix deposition was dose dependently elevated in the presence of ACTH. These data highlight that the melanocortin system promotes chondrocyte phenotype development and their differentiation into mature chondrocytes, leading to an elevation in intracellular free calcium[135]. (Table 2)

**TABLE 2**
**Melanocortin Peptide Effects in Arthritic Pathologies**

| Peptide Treatment | Observed Effects | Ref. |
|---|---|---|
| α-MSH | Modulates arthritic weight loss, joint damage, swelling, and score | [130] |
| | Inhibits TNF-α–induced, MMP-13–induced chondrocyte damage | [131] |
| $ACTH_{1-39}$ | Improves joint pain, swelling, and rheumatic symptoms in patients with RA | [19,20] |
| | Clinically effective in patients with gout | [136,138] |
| $ACTH_{4-10}$ | Modulates neutrophil migration and proinflammatory cytokines in a corticosterone-independent fashion, effects blocked by $MC_3$ anatagonist SHU9119 | [49] |
| $\gamma_2$-MSH | Modulates neutrophil migration and proinflammatory cytokines effects blocked by $MC_3$ antagonist SHU9119 | [50] |
| MTII | Modulates neutrophil migration and proinflammatory cytokines effects blocked by $MC_3$ antagonist SHU9119 | [67] |
| | Induces anti-inflammatory proteins IL-10 and HO-1 | [52] |
| dTRP$^8$-$\gamma_2$-MSH | Modulates neutrophil migration and proinflammatory cytokines effects blocked by $MC_3$ antagonist SHU9119 in recessive yellow e/e mouse | [79] |
| | Inhibits neutrophil migration and cytokine production in C57 mice, but not $MC_3$ null mice | [121] |

## Gouty Arthritis

Gouty arthritis is characterised by the deposition of urate crystals into the intra-articular and periarticular space, leading to production of chemokines and infiltrating neutrophils. Melanocortins were first shown to have biological efficacy in treating gout in the 1950s with the parent hormone $ACTH_{1-39}$ being used clinically, although no mechanism of action was described for their effects[136]. Long-term $ACTH_{1-39}$ administration does, however, lead to a number of side effects, including suppression of the HPA axis, and thus its use was superseded by other therapies. Current treatments for gout focus largely on the use of NSAIDs, which cause a number of side effects, including peptic ulceration, gastric problems, and are poorly tolerated in the elderly and patients with renal insufficiency. Glucocorticoids are also used when NSAIDs are deemed risky or contraindicated, but a number of side effects, including osteoporosis, can be observed. Colchicine treatment derived from the autumn crocus is also used to suppress the inflammatory process and reduce neutrophil infiltration, but side effects include profuse diarrhoea, nausea, vomiting, and gastrointestinal haemorrhage, which limits patient compliability[137]. During the last 15 years, a renewed interest in the use of $ACTH_{1-39}$ has occurred, demonstrating a greater efficacy than conventional glucocorticoids in controlling gout[138]. The authors suggested the existence of another mechanism of action other than the well-characterised stimulation of ACTH/$MC_2$ expressed on the adrenal gland[138]. Therefore, anti-inflammatory fragments of $ACTH_{1-39}$ that do not activate the HPA axis have been sought in the hope that they may have a better safety profile. Utilising murine models of gout, it has been demonstrated that smaller fragments of the POMC gene, such as α-MSH, β-MSH, and $ACTH_{4-10}$, could reduce urate crystal–induced neutrophil migration, and proinflammatory cytokine and chemokine release[49]. Of particular interest was the observation that the anti-inflammatory effects of these peptides occurred in a corticosterone-independent manner and thus no reflex stimulation of the HPA axis occurred. These findings mirror those observed for $ACTH_{4-10}$ in rat skin inflammation, where it was shown to inhibit prostaglandin generation and oedema formation[139]. The biological efficacy of these peptides was shown to occur via $MC_3$ expression on peritoneal macrophages with subsequent elevations in cAMP. Further confirmation of the role of $MC_3$ in mediating the anti-inflammatory effects of these peptides was highlighted when pretreatment of mice with the $MC_3/_4$ antagonist SHU9119 led to an attenuation of their efficacy. Based on these findings, a hypothesis was proposed that $MC_3$ could be a novel therapeutic target for modulating the anti-inflammatory effects of the melanocortins, at least in this model of monosodium urate crystal deposition[49]. Further studies promote support for this hypothesis with natural and synthetic agonists of the $MC_3$, $\gamma_2$-MSH[139] and MTII[67], respectively, attenuating parameters of inflammation both *in vivo* and *in vitro*[50]. In a rodent model developed to mimic certain aspects of the human pathology, urate crystals were injected into the knee joint, which led to migration of neutrophils preceded by the release of IL-1β and IL-6. Local and systemic administration of the parent hormone $ACTH_{1-39}$ resulted in significant inhibition of all parameters associated with joint inflammation (swelling, arthritic score, neutrophil migration). This inhibition occurred in a corticosterone-independent manner following local administration and was still efficacious in adrenelectomised rats. These data highlighted a role for a locally expressed MC confirmed by electron microscopy within the joint and that the effects of $ACTH_{1-39}$ were not mediated by adrenal gland $MC_2$ expression[140]. Given the anticytokine properties of the melanocortins, several studies have tried to identify whether these peptides could aid in the resolution of inflammation in these rodent models. Melanocortins were shown to induce the anti-inflammatory protein heme-oxygenase 1 both *in vitro* and *in vivo* following administration of the synthetic $MC_3/_4$ agonist MTII, an effect blocked by the HO-1 inhibitor zinc protoporphyrin IX[52]. A similar induction was noted for IL-10 by $ACTH_{1-39}$ or MTII, which occurred in a time-dependent fashion, was abrogated by the PKA inhibitor H-89, and only partially blocked by the p38 MAPK inhibitor SB203580[96]. Other support for a role for $MC_3$ comes from studies utilising the selective $MC_3$ agonist dTRP$^8$-γ-MSH, which exhibited a protective effect in mice with a nonfunctional $MC_1$ (recessive yellow (e/e) mouse)[79], but not in $MC_3$ null mice[121]. (Fig. 3)

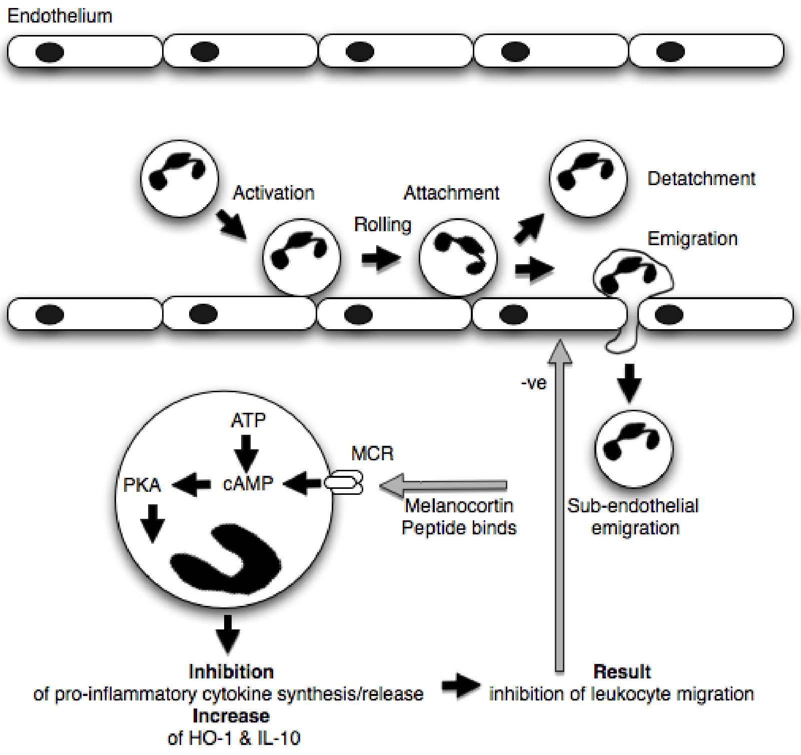

**FIGURE 3.** $MC_3$ proposed mechanism in modulating macrophage activation and subsequent inhibition of inflammatory arthritis. Resident peritoneal and knee joint MØ express the $MC_3$; agonism at this receptor leads to the accumulation of cAMP. This leads to the reduction in the synthesis and release of proinflammatory cytokines, with increases in anti-inflammatory mediators including HO-1. The net result is a reduction in the migration of leukocytes and an impact on the host inflammatory response.

### Current and Emerging Therapies

A vast array of novel therapies have been developed over the last decade or so for the treatment of arthritic pathologies, especially RA, which now sit alongside the more traditional approaches that include methotrexate, NSAIDs, and glucocorticoids. The development of these biologicals over the last 10 years and the recognition of the role played by more aggressive drugs, such as methotrexate, have led to positive results in the clinical management of patients[141]. The “new biologicals”, TNF-α binding proteins, including the chimeric monoclonal antibody infliximab (Remicade®) and TNF receptor (p75):FcIgG construct etanercept (Enbrel®), and the IL-1 antagonist anakinra, have begun to play an ever-increasing role in the management of arthritic pathologies[141]. Inflixmab administered by i.v. infusion exerts its biological effect by binding to TNF-α, thus reducing its effects in chronic inflammatory diseases including RA, Crohns disease, and ulcerative coltis. Whilst etanercept administered s.c. exerts its effects by preventing binding of TNF-α to its receptor and has been shown to be beneficial in RA, it can also be administered by the patient at home. These drugs have been invaluable in the clinical management of chronic arthritis and other inflammatory diseases, although increased risk of infections including tuberculosis (TB) have been reported with a three- to fourfold higher risk in patients taking infliximab compared to etanercept[142], thus careful monitoring of patients is required. A recent review by Wallis sought to identify whether or not the different isotypes lead to an elevated risk of infections. The data also suggest that TNF antibodies pose a greater risk than soluble TNF-α receptor antagonists. In part, this could be due to latent TB and so routine screening of individuals prior to placing them on this therapy could reduce the number of opportunistic infections being observed. This could work well in western markets, but as the drugs become used worldwide, prevention of TB will have to be undertaken prior to commencement of therapy[143]. Overall, these novel drugs play a pivotal role in the clinical management of chronic inflammatory diseases and, like all drugs, side effects occur and thus patients need to be carefully monitored to help prevent against the occurrence of serious complications. In development for RA are a number of cytokine antagonists, including those that target IL-6 (tocilizumab, which has been approved for use in Japan), IL-15, and RANKL, as well as the development of kinase inhibitors, including those that inhibit JAK-3 and SyK[141]. Although patients respond well to these agents, substantial numbers do not and so the development of novel therapies is essential, which may have a better safety profile with a reduced risk of opportunistic infections.

## CONCLUDING COMMENTS

Melanocortins have a vast repertoire of physiological and pharmacological actions. They have been proposed to play a role in modulating many disease pathologies, including inflammatory and cardiovascular disease, and are of major interest to the pharmaceutical industry for the treatment of metabolic disorders such as obesity. The generation of proinflammatory cytokines and the associated inflammatory responses are an essential part of the body’s defence mechanism to afford protection to an individual following exposure to foreign pathogens and injury, thus helping to restore homeostasis. Excessive and/or long-term activation of these processes, however, can lead to a number of chronic inflammatory pathologies, including the arthritides, and thus are detrimental to health[23]. Identification of endogenous modulators of the host inflammatory response, such as the melanocortins, represent exciting therapeutic targets for drug discovery and could potentially be used as a combination therapy with existing medication, thus targeting multiple aspects of the host inflammatory response as opposed to single pathways. This approach could have multiple benefits, including potentially reducing the side effects associated with current therapies. The melanocortins exert their effects at both ends of the inflammatory process, initially inhibiting the expression of proinflammatory cytokines and adhesion molecules, and, at later time points, increasing the levels of anti-inflammatory proteins to ensure resolution of the host inflammatory response. They are an exciting therapeutic target for drug discovery

(Fig. 4). In some respects, melanocortin activity resembles the "sledgehammer" properties of a steroid, without the side effects, but given their short half-life, it is unclear whether they could be first-line therapeutics. The development of orally active, small molecule MC agonists, however, may lead to useful first-line therapeutics and the lack of accumulation within the body normally associated with peptide therapy could result in reduced side effects compared to existing treatments[31]. Another exciting possibility is the potential identification of tissue-specific receptor subtypes allowing for a more directed pharmacological intervention or the development of dual inhibitors directed at $MC_1$ and $MC_3$. The net result of such a strategy would be an enhanced inhibition of leukocyte migration to the site of tissue injury and potentially lower doses of both agonists, thus having the potential to reduce toxic side effects. What is clear is that we are entering an era where harnessing the body's own natural defences could lead to novel therapeutics, as demonstrated by a host of other anti-inflammatory peptides including annexin 1[144] and galectin[145].

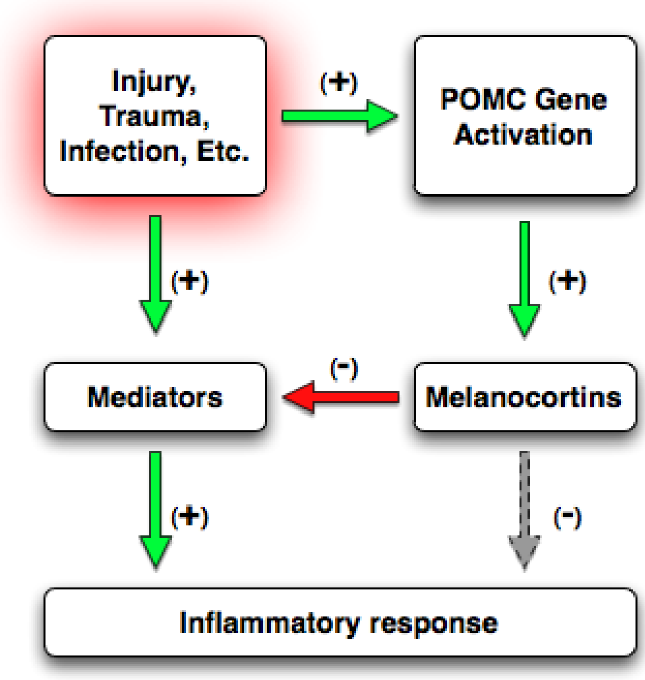

**FIGURE 4.** Melanocortins modulate the host-inflammatory response. Injury, trauma, and infection lead to the release of biochemical mediators that drive the inflammatory response leading to tissue damage. As a counter-regulatory mechanism, these causative agents lead to activation of the POMC gene and subsequent melanocortin peptide production. The melanocortin peptides, including α-MSH, lead to a reduction in the release of proinflammatory mediators and lead to a resolution of the inflammatory response.

## ACKNOWLEDGEMENTS

MK is supported by Research Committee, University of Westminster. (Grant Code CJ_Kan); HBP is a postdoctoral scientist supported by the Arthritis Research Campaign (Grant Code 18049).

## References

1. Bajekal, M., Osborne, V., Yar, M., and Meltzer, H. (2006) Focus on Health. Office of National Statistics, U.K.
2. March, L.M. and Bachmeier, C.M.J. (1997) Economics of osteoarthritis: a global perspective. *Baillieres Clin. Rheumatol.* **11**, 817–834.
3. Buckwalter, J.A. and Mankin, H.J. (1997) Articular cartilage part II: degeneration and osteoarthrosis, repair, regeneration and transplantation. *J. Bone Joint Surg. Am.* **79-A**, 612–632.
4. Kim, H.A. and Blanco, F.J. (2007) Cell death and apoptosis in osteoarthritic cartilage. *Curr. Drug Targets* **8**, 333–345.
5. Martin, J.A. and Buckwalter, J.A. (2002) Aging, articular cartilage chondrocyte senescence and osteoarthritis. *Biogerentology* **3**, 257–264.
6. Mobasheri, A. (2002) Role of chondrocyte death and hypocellularity in ageing human articular cartilage and the pathogenesis of osteoarthritis. *Med. Hypotheses* **58**, 193–197.
7. Lee, D.M. and Weinblatt, M.E. (2001) Rheumatoid arthritis. *Lancet* **358**, 903–911.
8. Brennan, F.M. and McInnes, I.B. (2008) Evidence that cytokines play a role in rheumatoid arthritis. *J. Clin. Invest.* **118**, 3537–3545.
9. van Venrooij, W.J, van Beers, J.J.B.C., and Pruijn, G.J.M. (2008) Anti-CCP antibody, a marker for the early detection of rheumatoid arthritis. *Ann. N. Y. Acad. Sci.* **1143**, 268–285.
10. Cronstein, B.N. and Terkeltaub, R. (2006) The inflammatory process of gout and its treatment. *Arthritis Res. Ther.* **8(Suppl. 1),** S3.
11. So, A. (2008) Developments in the scientific and clinical understanding of gout. *Arthritis Res. Ther.* **10,** 221–227.
12. Morgan, C., Lunt, M., Brightwell, H., Bradburn, P., Fallow, W., Lay, M., Silman, A., and Bruce, I.N. (2003) Contribution of patient related differences to multidrug resistance in rheumatoid arthritis. *Ann. Rheum. Dis.* **62(1)**, 15–19.
13. Midwood, K., Sacre, S., Piccinini, A.M., Inglis, J., Trebaul, A., Chan, E., Drexler, S., Sofat, N., Kashiwagi, M., Orend, G., Brennan, F., and Foxwell, B. (2009) Tenascin-C is an endogenous activator of Toll-like receptor 4 that is essential for maintaining inflammation in arthritic joint disease. *Nat. Med.* **15(7),** 774–780.
14. Nakagawa, S., Arai, Y., Mori, H., Matsushita, Y., Kubo, T., and Nakanishi, T. (2009) Small interfering RNA targeting CD81 ameliorated arthritis in rats. *Biochem. Biophys. Res. Commun.* **388(3),** 467–472.
15. Doblado, M.A. and Moley, K.H. (2009) Facilitative glucose transporter 9 (GLUT9), a unique hexose and urate transporter. *Am. J. Physiol. Endocrinol. Metab.* **297(4),** E831–835.
16. Torres, R., Macdonald, L., Croll, S.D., Reinhardt, J., Dore, A., Stevens, S., Hylton, D.M., Rudge, J.S., Liu-Bryan, R., Terkeltaub, R.A., Yancopoulos, G.D., and Murphy, A.J. (2009) Hyperalgesia, synovitis, and multiple biomarkers of inflammation are suppressed by IL-1 inhibition in a novel animal model of gouty arthritis. *Ann. Rheum. Dis.* **68(10),** 1602–1608.
17. Rudolphi, K., Gerwin, N., Verziji, N., van der Kraan, P., and van den Berg, W. (2003) Praincasan, an inhibitor of interleukin-1 beta converting enzyme, reduces joint damage in two murine models of osteoarthritis. *Osteo Cart.* **11(10),** 738–746.
18. Murase, A., Okumura, T., Sakakibara, A., Tonai-Kachi, H., and Takada, J. (2008) Effect of prostanoid EP4 receptor antagonist CJ-042,794, in rat models of pain and inflammation. *Eur. J. Pharmacol.* **580(1–2),** 116–121.
19. Hench, P.S. and Kendall, E.C. (1949) The effect of a hormone of the adrenal cortex (17-hydroxy-11-dehydrocorticosterone; compound E) and of pituitary adrenocorticotropic hormone on rheumatoid arthritis. *Mayo Clin. Proc.* **24(8)**, 181–197.
20. Hench, P.S., Kendall, E.C., Slocumb, C.H., and Polley, H.F. (1949) Adrenocortical hormone in arthritis: preliminary report. *Ann. Rheum. Dis.* **8(2)**, 97–104.
21. Heinig, J.A., Keeley, F.W., Robson, P., Sower, S.A., and Youson, J.H. (1995) The appearance of proopiomelanocortin early in vertebrate evolution: cloning and sequencing of POMC from a Lamprey pituitary cDNA library. *Gen. Comp. Endocrinol.* **99**, 137–144.
22. Lipton, J.M. and Catania, A. (1997) Anti-inflammatory actions of the neuroimmunomodulator alpha-MSH. *Immunol. Today* **18,** 140–145.
23. Getting, S.J. (2006) Targeting melanocortin receptors for the treatment of disease pathologies. *Pharmacol. Ther.* **111(1)**, 1–15.
24. Takahashi, H., Teranishi, Y., Nakanishi, S., and Numa, S. (1981) Isolation and structural organization of the human corticotropin-beta-lipotropin precursor gene. *FEBS Lett.* **135,** 97–102.
25. Uhler, M. and Herbert, E. (1983) Complete amino acid sequence of mouse pro-opiomelanocortin derived from the nucleotide sequence of pro-opiomelanocortin cDNA. *J. Biol. Chem.* **258,** 257–261.
26. Drouin, J. and Goodman, H.M. (1980) Most of the coding region of rat ACTH beta-LPH precursor gene lacks intervening sequences. *Nature* **288,** 610–613.
27. Castro, M.G. and Morrison., E. (1997) Post-translational processing of proopiomelanocortin in the pituitary and in the brain. *Crit. Rev. Neurobiol.* **11(1),** 35–57.

28. Benjannet, S., Rondeau, N., Day, R., Chretien, M., and Seidah, N.G. (1991) PC1 and PC2 are proprotein convertases capable of cleaving proopiomelanocortin at distinct pairs of basic residues. *Proc. Natl. Acad. Sci. U. S. A.* **88,** 3564–3568.
29. Bloomfield, G.A., Scott, A.P., Lowry, P.J., Gikes, J.J.H., and Rees, L.H. (1974) A reappraisal of human b-MSH. *Nature* **252,** 492–493.
30. Scott, A.P. and Lowry, P.J. (1974) Adrenocorticotropic and melanocyte-stimulating peptides in the human pituitary. *Biochem. J.* **139,** 593–602.
31. Catania, A., Gatti, S., Colombo, G., and Lipton, J.M. (2004) Targeting melanocortin receptors as a novel strategy to control inflammation. *Pharmacol. Rev.* **56,** 1–29.
32. Kelly, J.M., Moir, A.J., Carlson, K., Yang, Y., MacNeil, S., and Haycock, J.W. (2006) Immobilized alpha-melanocyte stimulating hormone 10-13 (GKPV) inhibits tumour necrosis factor alpha stimulated NF-kappaB activity. *Peptides* **27,** 431–437.
33. Manna, S.K. and Aggarwal, B.B. (1998) a-Melanocyte stimulating hormone inhibits the nuclear transcription factor NF-kB activation by various inflammatory agents. *J. Immunol.* **161,** 2873–2880.
34. Wikberg, J.E., Muceniece, R., Mandrika, I., Prusis, P., Lindblom, J., Post, C., and Skottner, A. (2000) New aspects on the melanocortins and their receptors. *Pharmacol. Res.* **42,** 393–420.
35. Chhajlani, V. and Wikberg, J.E.S. (1992) Molecular cloning and expression of the human melanocyte stimulating hormone receptor cDNA. *FEBS Lett.* **309(3),** 417–420.
36. Mountjoy, K.G., Robbins, L.S., Mortrud, M.T., and Cone, R.D. (1992) The cloning of a family of genes that encode the melanocortin receptors. *Science* **257,** 1248–1251.
37. Bhardwaj, R., Becher, E., Mahnke, K., Hartmeyer, M., Schwarz, T., Scholzen, T., and Luger, T.A. (1997) Evidence for the differential expression of the functional alpha-melanocyte-stimulating hormone receptor MC-1 on human monocytes. *J. Immunol.* **158,** 3378–3384.
38. Catania, A., Rajora, N., Capsooni, F., Minonzio, F., Star, R.A., and Lipton, J.M. (1996) The neuropeptide a-MSH has specific receptors on neutrophils and reduces chemotaxis in vitro. *Peptides* **17(4),** 675–679.
39. Hartmeyer, M., Scholzen, T., Becher, E., Bhardwaj, R.S., Schwarz, T., and Luger, T.A. (1997) Human dermal microvascular endothelial cells express the melanocortin receptor type 1 and produce increased levels of IL-8 upon stimulation with alpha-melanocyte-stimulating hormone. *J. Immunol.* **159,** 1930–1937.
40. Hill, R.P., MacNeil, S., and Haycock, J.W. (2006) Melanocyte stimulating hormone peptides inhbit TNF-alpha signaling in human dermal fibroblast cells. *Peptides* **27,** 421–430.
41. Adachi, S., Nakano, T., Vliagoftis, H., and Metcalfe, D.D. (1999) Receptor-mediated modulation of murine mast cell function by alpha-melanocyte stimulating hormone. *J. Immunol.* **163,** 3363–3368.
42. Neumann-Andersen, G., Nagaeva, O., Mandrika, I., Petrovska, R., Muceniece, R., Mincheva-Nilsson, L., and Wikberg, J.E. (2001) MC(1) receptors are constitutively expressed on leucocyte subpopulations with antigen presenting and cytotoxic functions. *Clin. Exp. Immunol.* **126,** 441–446.
43. Xia, Y., Wikberg, J.E., and Chhajlani, V. (1995) Expression of melanocortin 1 receptor in periaqueductal gray matter. *Neuroreport* **6,** 2193–2196.
44. Tatro, J.B. (1996) Receptor biology of the melanocortins, a family of neuroimmunomodulatory peptides. *Neuroimmunomodulation* **3(5)**, 259–284.
45. Penhoat, A., Jaillard, C., and Saez, J.M. (1989) Corticotropin positively regulates its own receptors and cAMP response in cultured bovine adrenal cells. *Proc. Natl. Acad. Sci. U. S. A.* **86,** 4978–4981.
46. Liakos, P., Chambaz, E.M., Feige, J.J., and Defaye, G. (1998) Expression of ACTH receptors (MC2-R and MC5-R) in the glomerulosa and the fasciculata-reticularis zones of bovine adrenal cortex. *Endocr. Res.* **24,** 427–432.
47. Gantz, I., Konda, Y., Tashiro, T., Shimoto, Y., Miwa, H., Munzert, G., Watson, S.J., DelValle, J., and Yamada, T. (1993) Molecular cloning of a novel melanocortin receptor. *J. Biol. Chem.* **268(11),** 8246–8250.
48. Butler, A.A., Kesterson, R.A., Khong, K., Cullen, M.J., Pelleymounter, M.A., Dekoning, J., Baetscher, M., and Cone, R.D. (2000) A unique metabolic syndrome causes obesity in the melanocortin-3 receptor-deficient mouse. *Endocrinology* **141,** 3518–3521.
49. Getting, S.J., Gibbs, L., Clark, A.J.L., Flower, R.J., and Perretti, M. (1999) POMC gene derived peptides activate melanocortin type 3 receptor on murine macrophages, suppress cytokine release and inhibit neutrophil migration in acute experimental inflammation. *J. Immunol.* **162,** 7446–7453.
50. Getting, S.J., Allcock, G.H., Flower, R., and Perretti, M. (2001) Natural and synthetic agonists of the melanocortin receptor type 3 possess anti-inflammatory properties. *J. Leukoc. Biol.* **69,** 98–104.
51. Getting, S.J., Christian, H.C., Lam, C.W., Gavins, F.N., Flower, R.J., Schioth, H.B., and Perretti, M. (2003) Redundancy of a functional melanocortin 1 receptor in the anti-inflammatory actions of melanocortin peptides: studies in the recessive yellow (e/e) mouse suggest an important role for melanocortin 3 receptor. *J. Immunol.* **170,** 3323–3330.
52. Lam, C.W., Getting, S.J., and Perretti, M. (2005) In vitro and in vivo induction of heme oxygenase 1 in mouse macrophages following melanocortin receptor activation. *J. Immunol.* **174,** 2297–2304.
53. Guarini, S., Schioth, H.B., Mioni, C., Cainazzo, M., Ferrazza, G., Giuliani, D., Wikberg, J.E., Bertolini, A., and Bazzani, C. (2002) MC(3) receptors are involved in the protective effect of melanocortins in myocardial ischemia/reperfusion-induced arrhythmias. *Naunyn Schmiedebergs Arch. Pharmacol.* **366,** 177–182.

54. Getting, S.J., Di Filippo, C., Christian, H.C., Lam, C.W., Rossi, F., D'Amico, M., and Perretti, M. (2004) MC-3 receptor and the inflammatory mechanisms activated in acute myocardial infarct. *J. Leukoc. Biol.* **76,** 845–853.
55. Alvaro, J.D., Tatro, J.B., Quillan, J.M., Fogliano, M., Elsenhard, M., Lerner, M.R., Nestler, E.J., and Duman, R.S. (1996) Morphine down regulates melanocortin 4 receptor expression in brain regions that mediate opiate addiction. *Mol. Pharmacol.* **50,** 583–591.
56. Mountjoy, K.G., Mrtrud, M.T., Low, M.J., Simerly, R.B., and Cone, R.D. (1994) Localization of the melanocortin-4 receptor (MC4-R) in neuroendocrine and autonomic control circuits in the brain. *Mol. Endocrinol.* **8(10),** 1298–1308.
57. Gantz, I., Miwa, H., Konda, Y., Shimoto, Y., Tashiro, T., Watson, S.J., DelValle, J., and Yamada, T. (1993) Molecular cloning, expression, and gene localization of a fourth melanocortin receptor. *J. Biol. Chem.* **268(20),** 15174–15179.
58. Martin, W.J. and MacIntyre, D.E. (2004) Melanocortin receptors and erectile function. *Eur. Urol.* **45,** 706–713.
59. Starowicz, K. and Przewlocka, B. (2003) The role of melanocortins and their receptors in inflammatory processes, nerve regeneration and nociception. *Life Sci.* **73,** 823–847.
60. Buggy, J.J. (1998) Binding of alpha-melanocyte-stimulating hormone to its G-protein-coupled receptor on B-lymphocytes activates the Jak/STAT pathway. *Biochem. J.* **331(Pt 1),** 211–216.
61. Taylor, A. and Namba, K. (2001) In vitro induction of CD25+ CD4+ regulatory T cells by the neuropeptide alpha-melanocyte stimulating hormone (alpha-MSH). *Immunol. Cell Biol.* **79,** 358–367.
62. Luger, T.A., Scholzen, T.E., Brzoska, T., and Bohm, M. (2003) New insights into the functions of alpha-MSH and related peptides in the immune system. *Ann. N. Y. Acad. Sci.* **994,** 133–140.
63. Gantz, I., Shimoto, Y., Konda, Y., Miwa, H., Dickinson, C.J., and Yamada, T. (1994) Molecular cloning, expression, and characterization of a fifth melanocortin receptor. *Biochem. Biophys. Res. Commun.* **200(3)**, 1214–1220.
64. Schioth, H.B., Mutulis, F., Muceniece, R., Prusis, P., and Wikberg, J.E.S. (1998) Discovery of novel melanocortin 4 receptor selective MSH analogues. *Br. J. Pharmacol.* **124,** 75–82.
65. Sawyer, T.K., Sanfilippo, P.J., Hruby, V.J., Engel, M.H., Heward, C.B., Burnett, J.B., and Hadley, M.E. (1980) 4-Norleucine, 7-D-phenylalanine-alpha-melanocyte-stimulating hormone: a highly potent alpha-melanotropin with ultralong biological activity. *Proc. Natl. Acad. Sci. U. S. A.* **77,** 5754–5758.
66. Al-Obeidi, F., Castrucci, A.M., Hadley, M.E., and Hruby, V.J. (1989) Potent and prolonged acting cyclic lactam analogues of alpha-melanotropin: design based on molecular dynamics. *J. Med. Chem.* **32,** 2555–2561.
67. Fan, W., Boston, B.A., Kesterson, R.A., Hruby, V.J., and Cone, R.D. (1997) Role of melanocortinergic neurons in feeding and the agouti obesity syndrome. *Nature* **385,** 165–168.
68. Grieco, P., Han, G., Weinberg, D., Van der Ploeg, L.H., and Hruby, V.J. (2002) Design and synthesis of highly potent and selective melanotropin analogues of SHU9119 modified at position 6. *Biochem. Biophys. Res. Commun.* **292,** 1075–1080.
69. Martignoni, E., Godi, L., Pacchetti, C., Berardesca, E., Vignoli, G.P., Albani, G., Mancini, F., and Nappi, G. (1997) Is seborrhea a sign of autonomic impairment in Parkinson’s disease? *J. Neural Trans.* **104,** 1295–1304.
70. Brzoka, T., Kalden, D.H., Fastrich, M., Möller, M., Schiöth, H.B., Wikberg, J.E.S., and Luger, T. (1999) Two new α-melanocyte stimulating (α-MSH) analogues (MS05 and MS09) are potent immunomodulators in vivo and in vitro. *J. Invest. Dermatol.* **113,** 482.
71. Ignar, D.M., Andrews, J.L., Jansen, M., Eilert, M.M., Pink, H.M., Lin, P., Sherrill, R.G., Szewczyk, J.R., and Conway, J.G. (2003), Regulation of TNF-α secretion by a specific melanocortin-1 receptor peptide agonist. *Peptides* **24,** 709–716.
72. Hruby, V.J., Lu, D., Sharma, S.D., Castrucci, A.L., Kesterson, R.A., Al-Obeidi, F.A., Hadlley, M.E., and Cone, R.D. (1995) Cyclic lactam a-melanotropin analogues of NDP-a-MSH with bulky aromatic amino acids at position 7 show high antagonist potency and selectivity at specific melanocortin receptors. *J. Med. Chem.* **38,** 3454–3461.
73. Kask, A., Rago, L., Mutulis, F., Pahkla, R., Wikberg, J.E., and Schioth, H.B. (1998) Selective antagonist for the melanocortin 4 receptor (HS014) increases food intake in free-feeding rats. *Biochem. Biophys. Res. Commun.* **245,** 90–93.
74. Lu, D., Willard, D., Patel, I.R., Kadwell, S., Overton, L., Kost, T., Luther, M., Wenbiao, C., Woychik, R.P., Wilkison, W.O., and Cone, R.D. (1994) Agouti protein is an antagonist of the melanocyte stimulating-hormone receptor. *Nature* **371,** 799–802.
75. Wilson, B.D., Ollmann, M.M., Kang, L., Stoffel, M., Bell, G.I., and Barsh, G.S. (1995) Structure and function of ASP, the human homolog of the mouse agouti gene. *Hum. Mol. Gen.* **4(2)**, 223–230.
76. Voisey, J. and van Daal, A. (2002) Agouti: from mouse to man, from skin to fat. *Pigment Cell Res.* **15(1)**, 10–18.
77. Ollmann, M.M., Wilson, B.D., Yang, Y.K., Kerns, J.A., Chen, Y., Gantz I., and Barsh, G.S. (1997) Antagonism of central melanocortin receptors in vitro and in vivo by agouti-related protein. *Science* **278,** 135–138.
78. Fong, T.M., Mao, C., MacNeil T., Kalyani R., Smith T., Weinberg D., Tota, M.R., and Van der Ploeg, L.H.T. (1997) ART (protein product of agouti-related transcript) as an antagonist of MC-3 and MC-4 receptors. *Biochem. Biophys. Res. Commun.* **237,** 629–631.
79. Getting, S.J., Lam, C.W., Leoni, G., Gavins, F.N., Grieco, P., and Perretti M. (2006) [D-TRP$^{8}$]-gamma-melanocyte stimulating hormone exhibits anti-inflammatory efficacy in mice bearing a non-functional MC1R (recessive yellow e/e mouse). *Mol. Pharmacol.* **70(6),** 1850–1855,

80. Holder, J.R. and Haskell-Luevano, C. (2004) Melanocortin ligands: 30 years of structure-activity relationship (SAR) studies. *Med. Res. Rev.* **24,** 325–356.
81. Sebhat, I.K., Martin, W.J., Ye, Z., Barakat, K., Mosley, R.T., Johnston, D.B., Bakshi, R., Palucki, B., Weinberg, D.H., MacNeil, T., Kalyani, R.N., Tang, R., Stearns, R.A., Miller, R.R., Tamvakopoulos, C., Strack, A.M., McGowan, E., Cashen, D.E., Drisko, J.E., Hom, G.J., Howard, A.D., MacIntyre, D.E., van der Ploeg, L.H., Patchett, A.A., and Nargund, R.P. (2002) Design and pharmacology of N-[(3R)-1,2,3,4-tetrahydroisoquinolinium- 3-ylcarbonyl]-(1R)-1-(4-chlorobenzyl)- 2-[4-cyclohexyl-4-(1H-1,2,4-triazol- 1-ylmethyl)piperidin-1-yl]-2-oxoethylamine (1), a potent, selective, melanocortin subtype-4 receptor agonist. *J. Med. Chem.* **45,** 4589–4593.
82. Pogozheva, I.D., Chai, B.X., Lomize, A.L., Fond, T.M., Weinberg, D.H., Nargund, R.P., Mulholland, M.W., Gantz, I., and Mosberg, H.I. (2005) Interactions of human melanocortin 4 receptor with non peptide and peptide agonists. *Biochemistry* **44(34),** 11329–11341.
83. Van der Ploeg, L.H., Martin, W.J., Howard, A.D., Nargund, R.P., Austin, C.P., Guan, X., Drisko, J., Cashen, D., Sebhat, I., Patchett, A.A., Figueroa, D.J., DiLella, A.G., Connolly, B.M., Weinberg, D.H., Tan, C.P., Palyha, O.C., Pong, S.S., MacNeil, T., Rosenblum, C., Vongs, A., Tang, R., Yu, H., Sailer, A.W., Fong, T.M., Huang, C., Tota, M.R., Chang, R.S., Stearns, R., Tamvakopoulos, C., Christ, G., Drazen, D.L., Spar, B.D., Nelson, R.J., and MacIntyre, D.E. (2002) A role for the melanocortin 4 receptor in sexual function. *Proc. Natl. Acad. Sci. U. S. A.* **99,** 11381–11386.
84. Allard, J., Reynolds, D.S., and Edmunds, N.J. (2008) Potentiation of reflex erectile responses in the anaesthetized rat by the selective melanocortin receptor 4 agonist MB243. *BJU Int.* **102(8),** 1029–1033.
85. Pontillo, J., Tran, J.A., Markison, S., Joppa, M., Fleck, B.A., Marinkovic, D., Arellano, M., Tucci, F.C., Lanier, M., Nelson, J., Sauders, J., Hoare, S.R.J., Foster, A.C., and Chen, C. (2005) A potent and selective nonpeptide antagonist of the melanocortin-4 receptor induces food intake in satiated mice. *Bioorg. Med. Chem. Lett.* **15(10),** 2541–2546.
86. Tucci, F.C., White, N.S., Markison, S., Joppa, M., Tran, J.A., Fleck, B.A., Madan, A., Dyck, B.P., Parker, J., Pontillo, J., Arellano, M., Marinkovic, D., Jiang, W., Chen, C.W., Gogas, K.R., Goodfellow, V.S., Saunders, J., Foster, A.C., and Chen, C. (2005) Potent and orally active non-peptide antagonists of the human melanocortin-4 receptor based on a series of trans-2-disubstituted cyclohexylpiperazines. *Bioorg. Med. Chem. Lett.* **15(19),** 4389–4395.
87. Herpin, T.F., Yu, G., Carlson, K.E., Morton, G.C., Wu, X., Kang, L., Tuerdi, H., Khanna, A., Tokarski, J.S., Lawrence, R.M., and Macor, J.E. (2003) Discovery of tyrosine-based potent and selective melanocortin-1 receptor small-molecule agonists with anti-inflammatory properties. *J. Med. Chem.* **46,** 1123–1126.
88. Smalley, K. and Eisen, T. (2000) The involvement of p38 mitogen-activated protein kinase in the alpha-melanocyte stimulating hormone (alpha-MSH)-induced melanogenic and anti-proliferative effects in B16 murine melanoma cells. *FEBS Lett.* **476,** 198–202.
89. Mandrika, I., Muceniece, R., and Wikberg, J.E. (2001) Effects of melanocortin peptides on lipopolysaccharide/interferon-gamma-induced NF-kappaB DNA binding and nitric oxide production in macrophage-like RAW 264.7 cells: evidence for dual mechanisms of action. *Biochem. Pharmacol.* **61(5),** 613–621.
90. Kapas, S., Purbrick, A., and Hinson, J.P. (1995) Role of tyrosine kinase and protein kinase C in the steroidogenic actions of angiotensin II, alpha-melanocyte-stimulating hormone and corticotropin in the rat adrenal cortex. *Biochem. J.* **305(Pt 2),** 433–438.
91. Englaro, W., Rezzonico, R., Durand-Clement, M., Lallemand, D., Ortonne, J.P., and Ballotti, R. (1995) Mitogen-activated protein kinase pathway and AP-1 are activated during cAMP-induced melanogenesis in B-16 melanoma cells. *J. Biol. Chem.* **270,** 24315–24320.
92. Ghosh, S. and Karin, M. (2002) Missing pieces in the NF-kappaB puzzle. *Cell* **109(Suppl),** S81–96.
93. Kalden, D.H., Scholzen, T., Brzoska, T., and Luger, T.A. (1999) Mechanisms of the antiinflammatory effects of alpha-MSH. Role of transcription factor NF-kappa B and adhesion molecule expression. *Ann. N. Y. Acad. Sci.* **885,** 254–261.
94. Ichiyama, T., Okada, K., Campbell, I.L., Furukawa, S., and Lipton, J.M. (2000) NF-kappaB activation is inhibited in human pulmonary epithelial cells transfected with alpha-melanocyte-stimulating hormone vector. *Peptides* **21,** 1473–1477.
95. Konda, Y., Gantz, I., DelValle, J., Shimoto, Y., Miwa, H., and Yamada, T. (1994) Interaction of dual intracellular signaling pathways activated by the melanocortin-3 receptor. *J. Biol. Chem.* **269,** 13162–13166.
96. Lam, C.W., Perretti, M., and Getting, S.J. (2006) Melanocortin receptor signalling in RAW264.7 macrophage cell line. *Peptides* **27(2),** 404–412.
97. Bar-Ilan, A., Savion, N., Naveh, N., and Ilan, A.B. (1992) Alpha melanocyte stimulating hormone (alpha-MSH) enhances eicosanoid production by bovine retinal pigment epithelium. *Prostaglandins* **43,** 31–44.
98. Luger, T.A., Scholzen, T., and Grabbe, S. (1997) The role of alpha-melanocyte-stimulating hormone in cutaneous biology. *J. Invest. Dermatol. Symp. Proc.* **2,** 87–93.
99. Bohm, M., Schulte, U., Kalden, H., and Luger, T.A. (1999) Alpha-melanocyte-stimulating hormone modulates activation of NF-kappa B and AP-1 and secretion of interleukin-8 in human dermal fibroblasts. *Ann. N. Y. Acad. Sci.* **885**, 277–286.
100. de Wied, D. (1999) Behavioural pharmacology of neuropeptides related to melanocortins and the neurohypophyseal hormones. *Eur. J. Pharmacol.* **375,** 1–11.

101. Vrinten, D.H., Kalkman, C.J., Adan, R.A., and Gispen, W.H. (2001) Neuropathic pain: a possible role for the melanocortin system? *Eur. J. Pharmacol.* **429,** 61–69.
102. Starowicz, K. and Przewlocka, B. (2003) The role of melanocortins and their receptors in inflammatory processes, nerve regeneration and nociception. *Life Sci.* **73,** 823–847.
103. Beckwith, B.E., Tinius, T.P., Hruby, V.J., al-Obeidi, F., Sawyer, T.K., and Affholter, J.A. (1989) The effects of structure-conformation modifications of melanotropin analogs on learning and memory: D-amino acid substituted linear and cyclic analogs. *Peptides* **10**, 361–368.
104. Klusa, V., Svirskis, S., Opmane, B., Muceniece, R., Skujins, A., Mutulis, F., Wikberg, J.E., and Schioth, H.B. (1998) Evaluation of behavioural effects of neural melanocortin receptor antagonists injected ICV and in VTA in rats. *Neuropeptides* **32,** 573–580.
105. Smolnik, R., Perras, B., Molle, M., Fehm, H.L., and Born, J. (2000) Event-related brain potentials and working memory function in healthy humans after single-dose and prolonged intranasal administration of adrenocorticotropin 4-10 and desacetyl-alpha-melanocyte stimulating hormone. *J. Clin. Psychopharmacol.* **20,** 445–454.
106. Schioth, H.B., Lagerstrom, M.C., Watanobe, H., Jonsson, L., Vergoni, A.V., Ringholm, A., Skarphedinsson, J.O., Skuladottir, G.V., Klovins, J., and Fredriksson, R. (2003) Functional role, structure, and evolution of the melanocortin-4 receptor. *Ann. N. Y. Acad. Sci.* **994,** 74–83.
107. Adan, R.A., Hillebrand, J.J., De Rijke, C., Nijenhuis, W., Vink, T., Garner, K.M., and Kas, M.J. (2003) Melanocortin system and eating disorders. *Ann. N. Y. Acad. Sci.* **994,** 267–274.
108. Ellacott, K.L. and Cone, R.D. (2004) The central melanocortin system and the integration of short- and long-term regulators of energy homeostasis. *Recent Prog. Horm. Res.* **59,** 395–408.
109. Luger, T.A., Brzoska, T., Scholzen, T.E., Kalden, D.H., Sunderkotter, C., Armstrong, C., and Ansel, J. (2000) The role of alpha-MSH as a modulator of cutaneous inflammation. *Ann. N. Y. Acad. Sci.* **917,** 232–238.
110. Rheins, L.A., Cotleur, A.L., Kleier, R.S., Hoppenjans, W.B., Saunder, D.N., and Nordlund, J.J. (1989) Alpha-melanocyte stimulating hormone modulates contact hypersensitivity responsiveness in C57/BL6 mice. *J. Invest. Dermatol.* **93,** 511–517.
111. Hiltz, M.E. and Lipton, J.M. (1990) Alpha-MSH peptides inhibit acute inflammation and contact sensitivity. *Peptides* **11,** 979–982.
112. Robertson, B., Dostal, K., and Daynes, R.A. (1988) Neuropeptide regulation of inflammatory and immunologic responses. The capacity of alpha-melanocyte-stimulating hormone to inhibit tumor necrosis factor and IL-1-inducible biologic responses. *J. Immunol.* **140,** 4300–4307.
113. Lipton, J.M., Ceriani, G., Macaluso, A., McCoy, D., Carnes, K., Biltz, J., and Catania, A. (1994) Antiinflammatory effects of the neuropeptide alpha-MSH in acute, chronic, and systemic inflammation. *Ann. N. Y. Acad. Sci.* **741,** 137–148.
114. Leoni, G., Patel, H.B., Sampaio, A.L., Gavins, F.N., Murray, J.F., Grieco, P., Getting, S.J., and Perretti, M. (2008) Inflamed phenotype of the mesenteric microcirculation of melanocortin type 3 receptor-null mice after ischemia-reperfusion. *FASEB J.* **22(12)**, 4228–4238.
115. Gatti, S., Colombo, G., Buffa, R., Turcatti, F., Garofalo, L., Carboni, N., Ferla, L., Fassati, L.R., Lipton, J.M., and Catania, A. (2002) alpha-Melanocyte-stimulating hormone protects the allograft in experimental heart transplantation. *Transplantation* **74,** 1678–1684.
116. Murphy, M.T., Richards, D.B., and Lipton, J.M. (1983) Antipyretic potency of centrally administered alpha-melanocyte stimulating hormone. *Science* **221,** 192–193.
117. Shih, S.T., Khorram, O., Lipton, J.M., and McCann, S.M. (1986) Central administration of alpha-MSH antiserum augments fever in the rabbit. *Am. J. Physiol.* **250,** R803–806.
118. Huang, Q.H., Entwistle, M.L., Alvaro, J.D., Duman, R.S., Hruby, V.J., and Tatro, J.B. (1997) Antipyretic role of endogenous melanocortins mediated by central melanocortin receptors during endotoxin-induced fever. *J. Neurosci.* **17,** 3343–3351.
119. Getting, S.J., Riffo-Vasquez, Y., Pitchford, S., Kaneva, M., Grieco, P., Page, C.P., Perretti, M., and Spina, D. (2008) A role for MC3R in modulating lung inflammation. *Pulm. Pharmacol. Ther.* **21(6),** 866–873.
120. Raap, U., Brzoska, T., Sohl, S., Path, G., Emmel, J., Herz, U., Braun, A., Luger, T., and Renz, H. (2003) Alpha-melanocyte-stimulating hormone inhibits allergic airway inflammation. *J. Immunol.* **171,** 353–359.
121. Getting, S.J., Lam, C.W., Chen, A.S., Grieco, P., and Perretti, M. (2006) Melanocortin 3 receptors control crystal-induced inflammation. *FASEB J.* **20(13),** 2234–2241.
122. Rajora, N., Boccoli, G., Catania, A., and Lipton, J.M. (1997) alpha-MSH modulates experimental inflammatory bowel disease. *Peptides* **18,** 381–385.
123. Oktar, B.K., Ercan, F., Yegen, B.C., and Alican, I. (2000) The effect of alpha-melanocyte stimulating hormone on colonic inflammation in the rat. *Peptides* **21,** 1271–1277.
124. Yoon, S.W., Lee, C.H., Kim, J.Y., Kim, J.Y., Sung, M.H., and Poo, H. (2008) Lactobacillus casei secreting alpha-MSH induces the therapeutic effect on DSS-induced acute colitis in Balb/c Mice. *J. Microbiol. Biotechnol.* **18(12),** 1975–1983.
125. Getting, S.J., Schioth, H.B., and Perretti, M. (2003) Dissection of the anti-inflammatory effect of the core and C-terminal (KPV) {alpha}-melanocyte stimulating hormone peptides. *J. Pharmacol. Exp. Ther.* **306,** 631–637.

126. Kannengiesser, K., Maaser, C., Heidemann, J., Luegering, A., Ross, M., Brzoska, T., Bohm, M., Luger, T.A., Domschke, W., and Kucharzik, T. (2008) Melanocortin-derived tripeptide KPV has anti-inflammatory potential in murine models of inflammatory bowel disease. *Inflamm. Bowel Dis.* **14(3)**, 324–331.
127. Colombo, G., Buffa, R., Bardella, M.T., Garofalo, L., Carlin, A., Lipton, J.M., and Catania, A. (2002) Anti-inflammatory effects of alpha-melanocyte-stimulating hormone in celiac intestinal mucosa. *Neuroimmunomodulation* **10,** 208–216.
128. Catania, A., Gerloni, V., Procaccia, S., Airaghi, L., Manfredi, M.G., Lomater, C., Grossi, L., and Lipton, J.M. (1994) The anticytokine neuropeptide alpha-melanocyte-stimulating hormone in synovial fluid of patients with rheumatic diseases: comparisons with other anticytokine molecules. *Neuroimmunomodulation* **1,** 321–328.
129. Star, R.A., Rajora, N., Huang, J., Stock, R.C., Catania, A., and Lipton, J.M. (1995) Evidence of autocrine modulation of macrophage nitric oxide synthase by a-melanocyte-stimulating hormone. *Proc. Natl. Acad. Sci. U. S. A.* **92,** 8016–8020.
130. Ceriani, G., Diaz, J., Murphree, S., Catania, A., and Lipton, J.M. (1994) The neuropeptide alpha-melanocyte stimulating hormone inhibits experimental arthritis in rats. *Neuroimmunomodulation* **1,** 28–32.
131. Yoon, S.W., Chun, J.S., Sung, M.H., Kim, J.Y., and Poo, H. (2008) alpha-MSH inhibits TNF-alpha-induced matrix metalloproteinase-13 expression by modulating p38 kinase and nuclear factor kappaB signaling in human chondrosarcoma HTB-94 cells. *Osteoarthritis Cartilage* **16(1),** 115–124.
132. Zhong, Y. and Bellamkonda, R.V. (2005) Controlled release of anti-inflammatory agent α-MSH from neural implants. *J. Control. Release* **106(3)**, 309–318.
133. Takaishi, H., Kimura, T., Dalal, S., Okada, Y., and D'Armiento, J. (2008) Joint diseases and matrix metalloproteinases: a role for MMP-13. *Curr. Pharm. Biotechnol.* **9(1),** 47–54.
134. Evans, J.F., Niu, Q.T., Canas, J.A., Shen, C.L., Aloia, J.F., and Yeh, J.K. (2004) ACTH enhances chondrogenesis in multipotential progenitor cells and matrix production in chondrocytes. *Bone* **35(1),** 96–107.
135. Evans, J.F., Shen, C.L., Pollack, S., Aloia, J.F., and Yeh, J.K. (2005) Adrenocorticotropin evokes transient elevations in intracellular free calcium ([Ca2+]i) and increases basal[Ca2+]i in resting chondrocytes through a phospholipase C-dependent mechanism. *Endocrinology* **146(7),** 3123–3132.
136. Gutman, A.B. and Yu, T.F. (1950) Effects of adrenocorticotropic hormone (ACTH) in gout. *Am. J. Med.* **9(1),** 24–30.
137. Ben-Chetrit, E. and Levy, M. (1998) Colchicine: 1998 update. *Semin. Arthritis Rheum.* **28,** 48–59.
138. Ritter, J., Kerr, L.D., Valeriano-Marcet, J., and Spiera, H. (1994) ACTH revisited: effective treatment for acute crystal induced synovitis in patients with multiple medical problems. *J. Rheumatol.* **21(4)**, 696–699.
139. Gecse, A., Ottlecz, A., Farago, M., Forster, T., and Telegdy, G. (1980) Effect of ACTH on prostaglandin induced vascular permeability. *Acta Physiol. Acad. Sci. Hung.* **55(4),** 305–311.
140. Getting, S.J., Christian, H.C., Flower, R.J., and Perretti, M. (2002) Activation of melanocortin type 3 receptor as a molecular mechanism for adrenocorticotropic hormone efficacy in gouty arthritis. *Arthritis Rheum.* **46,** 2765–2775.
141. Bingham, C.O., 3rd (2008) Emerging therapeutics for rheumatoid arthritis. *Bull. NYU Hosp. Jt. Dis.* **66(3),** 210–215.
142. Dixon, W.G., Hyrich, K.L., Watson, K.D., Lunt, M., Galloway, J., Ustianowski, A., Symmons, D.P. (2009) Drug-specific risk of tuberculosis in patients with rheumatoid arthritis treated with anti-TNF therapy: results from the British Society for Rheumatology Biologics Register (BSRBR). *Ann. Rheum. Dis.* [Epub ahead of print]
143. Wallis, R.S. (2009) Infectious complications of tumour necrosis factor blockade. *Curr. Opin. Infect. Dis.* **22(4),** 403–409.
144. Gavins, F.N., Leoni, G., and Getting, S.J. (2006) Annexin 1 and melanocortin peptide therapy for protection against ischaemic-reperfusion damage in the heart. *TheScientificWorldJOURNAL* **6,** 1008–1023.
145. Norling, L.V., Perretti, M., and Cooper, D. (2009) Endogenous galectins and the control of the host inflammatory response. *J. Endocrinol.* **201(2),** 169–184.
